# Convolutional autoencoder joint boundary and mask adversarial learning for fundus image segmentation

**DOI:** 10.3389/fnhum.2022.1043569

**Published:** 2022-12-06

**Authors:** Xu Zhang, Jiaqi Song, Chengrui Wang, Zhen Zhou

**Affiliations:** ^1^Department of Computer Science and Technology, Chongqing University of Posts and Technology, Chongqing, China; ^2^Key Laboratory of Tourism Multisource Data Perception and Decision, Ministry of Culture and Tourism, Chongqing, China; ^3^Chongqing Telecom System Integration Co., Ltd., Chongqing, China; ^4^Tianjin Eye Hospital, Tianjin, China; ^5^Tianjin Key Laboratory of Ophthalmology and Vision Science, Tianjin, China; ^6^Nankai University Affiliated Eye Hospital, Tianjin, China; ^7^Clinical College of Ophthalmology Tianjin Medical University, Tianjin, China

**Keywords:** optic disc and cup segmentation, unsupervised domain adaptation, convolutional autoencoder, adversarial learning, glaucoma screening

## Abstract

The precise segmentation of the optic cup (OC) and the optic disc (OD) is important for glaucoma screening. In recent years, medical image segmentation based on convolutional neural networks (CNN) has achieved remarkable results. However, many traditional CNN methods do not consider the cross-domain problem, i.e., generalization on datasets of different domains. In this paper, we propose a novel unsupervised domain-adaptive segmentation architecture called CAE-BMAL. Firstly, we enhance the source domain with a convolutional autoencoder to improve the generalization ability of the model. Then, we introduce an adversarial learning-based boundary discrimination branch to reduce the impact of the complex environment during segmentation. Finally, we evaluate the proposed method on three datasets, Drishti-GS, RIM-ONE-r3, and REFUGE. The experimental evaluations outperform most state-of-the-art methods in accuracy and generalization. We further evaluate the cup-to-disk ratio performance in OD and OC segmentation, which indicates the effectiveness of glaucoma discrimination.

## 1. Introduction

In the past few years, retinal fundus images have been used to diagnose retinal diseases. Glaucoma is a disease that causes damage to the optic nerve of the eye, resulting in decreased vision, but timely detection can further control the effect of glaucoma. Automated segmentation of the optic disc (OD) and optic cup (OC) in fundus images is helpful for the screening and diagnosis of glaucoma (Fu et al., 2018). The development of deep convolutional neural networks (CNNs) has significantly improved the automatic monitoring of OD and OC. However, traditional CNNs are mostly based on the assumption that the training (source) and testing (target) images have the same distribution. Since the fundus images are obtained from different patients and different imaging equipment, the distribution of the domains is not the same (Wang Z. et al., 2020). Some domain adaptive methods are applied to fundus image segmentation to reduce the distribution mismatch between the source domain and the target domain (Dou et al., 2018; Zhang et al., 2018). However, simple domain adaptation will lack generalization, so how to improve the generalization ability of the model is a problem worth discussing.

Recently, some data augmentation methods (Prakash et al., 2019; Yue et al., 2019; Zhou et al., 2020) used for domain generalization. Nevertheless, these methods are difficult to be extended for medical image segmentation problems due to the structured prediction characteristics of segmentation tasks (Wang S. et al., 2020). In the field of medical image analysis, some of the latest single-source domain generalization methods have explored various data augmentation techniques to improve the generalization ability of CNNs for medical image segmentation in other domains (Chen et al., 2020). At the same time, there is also a method (Zhang et al., 2022) that uses an improved GAN method to segment medical images. Nihal Zaaboub et al. proposed a method (Zaaboub et al., 2022) to localize OD, and vessels were extracted and eliminated. A novel Domain-oriented Feature Embedding (DoFE) framework (Wang S. et al., 2020) has been presented to improve the generalization ability of CNNs on unseen target domains by exploring the knowledge from multiple source domains. A BEAL framework (Wang et al., 2019a) proposed utilizes the adversarial learning to encourage the boundary prediction and mask the probability entropy map of the target domain to be similar to the source ones, generating more accurate boundaries and suppressing the high uncertainty predictions of OD and OC segmentation. Inspired by this boundary method, we propose to use a convolutional auto-encoder to augment the data, and perform adversarial learning on the boundary and entropy maps to generate more accurate boundaries for OD and OC segmentation.

**Figure 1:**
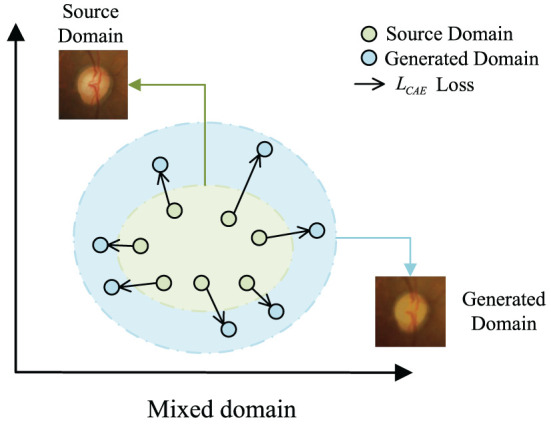
The motivation of *L*_*CAE*_. We expect to create out-of-domain augmentations by maximizing the loss *L*_*CAE*_.

In this work, we propose a novel domain adaptation framework, called Convolutional Autoencoder Joint Boundary and Mask Adversarial Learning (CAE-BMAL), to augment the data and improve OD/OC on the dataset segmentation accuracy. Our method is based on two main observations. First, as depicted in Figure 1, the convolutional autoencoder can generate an image with the same structure as the original image, but it is blurry than the original image, and some structures (such as blood vessels, etc.) have a certain degree of randomness which is helpful to learn the influence of different blood vessel orientations on OD/OC segmentation. This opens up the possibility to train and generalize on the source and target domains. Second, the influence of boundary segmentation also plays a vital role. The generated image has fuzzy boundaries, while the original image has clear boundaries. In this way, boundary segmentation can be performed more accurately on target domain datasets through adversarial learning of boundaries and guided masks. Based on these observations, we develop a boundary and mask adversarial learning based on the convolutional autoencoder method to segment the OD and OC from the target domain fundus images by generating more accurate boundaries and improving model generalization ability. The proposed method was extensively evaluated on three public fundus image datasets, i.e., Drishti-GS (Sivaswamy et al., 2015), RIM-ONE-r3 (Fumero et al., 2011), and REFUGE (Orlando et al., 2020), demonstrating state-of-the-art results. We also conducted an ablation study to show the effectiveness of each component in our method.

Our main contributions are summarized as follows: (1) We propose a new domain-adaptive segmentation framework that uses convolutional autoencoders to enhance source domain images to generate enhanced domains with the same semantics as the source domain images, but with different light and vessel shapes. Then they are put into the network to train together, in the segmentation process, the adaptive segmentation of the network under the influence of surrounding blood vessels is strengthened, and the generalization ability of the model is improved; (2) Our proposed network integrates the ideas of boundary prior and adversarial learning, and the two boundary subnetworks have their functions. Combined, the boundary discrimination can still show good segmentation performance in harsh conditions (such as dark light, and intricate blood vessels around the optic disc). (3) The proposed segmentation method is clinically meaningful in glaucoma screening based on extensive evaluations on three publicly available fundus image datasets.

## 2. Materials and methods

**Figure 2:**
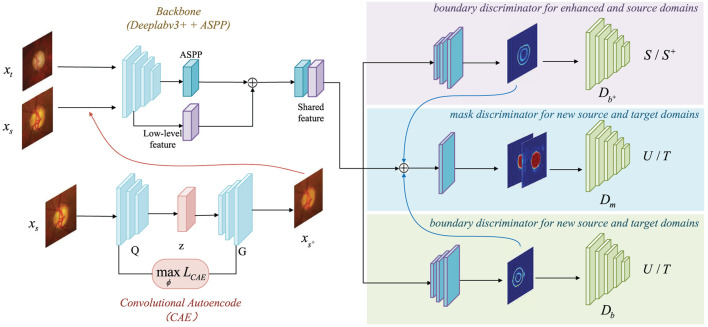
Overview of CAE-BMAL framework for domain daptation. The backbone is based on the DeepLabv3+ (Chen et al., 2018) architecture with Atrous Spatial Pyramid Pooling (ASPP) component followed by the adversarial learning branch structure which is used to classify.

The network architecture is exhibits in Figure 2. The key technical contribution of our method is a convolutional autoencoder-based boundary and mask adversarial learning framework, which uses both source and target domains to make accurate and confident predictions on the target domain while improving the generalization ability of the model. The proposed procedure is: (1) All source domain images are put into the CAE module for image generation, and all enhanced output images compose the enhanced domain. The enhanced output images retains the variety structure of blood vessels and the brightness with a rich semantic information; (2) The enhanced output images are put into the ASPP module together with the source domain and the target domain for feature extraction; (3) The finally extracted features are processed with three discriminators: *boundary discriminator for enhanced and source domains, mask discriminator for new source and target domains, boundary discriminator for new source and target domains*, as shown in Figure 2.

### 2.1. Convolutional autoencoder

How to generate images useful for fundus image segmentation has become a major challenge due to the time-consuming and labor-intensive manual labeling of fundus images and the rarity of labels. To solve this problem, we use a convolutional autoencoder to generate images similar to the original image but different in blood vessel structure and brightness. For the convenience of elaboration, we have added Table 1 as a comparison of mathematical symbols.

**Table 1:** Mathematical function symbol comparison table.

**Mathematics symbol**	**Meaning and explanation**
*X*_*S*_, *Y*_*S*_, *X*_*T*_, $X_{S^{+}}$, *X*_*U*_	Source domain, label domain of source domain, target domain, enhanced domain, union of source domain and enhanced domain (new source domain);
*x*_*s*_, $x_{s^{+}}$, *y*_*s*_	Image of source domain, image of augmented domain, label of image of source domain;
*Q*, *G*	Encoder function, decoder function;
$p_{x_{s}}^{m}$, $p_{x_{t}}^{m}$, $p_{x_{u}}^{m}$, $p_{x_{s}}^{b}$, $p_{x_{t}}^{b}$, $p_{x_{s^{+}}}^{b}$, $p_{x_{u}}^{b}$	Mask prediction for source domain images, mask prediction for target domain images, mask prediction for union images of source and enhanced domains, boundary prediction for source domain images, boundary prediction for target domain images, boundary prediction of enhanced domain images, boundary prediction for union images of source and enhanced domains;
*D*_*b*_, $D_{b^{+}}$, *D*_*m*_	Discriminator (see Section 2.2 for the corresponding function of the discriminator).

Formally, we aim at solving the problem of domain adaptation: A model is trained on a source domain $X_{S}\subset {\mathbb{R}}^{H\times W\times 3}$ along with ground truth segmentation maps $Y_{S}\subset {\mathbb{R}}^{H\times W}$, and a target domain $X_{T}\subset {\mathbb{R}}^{H\times W\times 3}$ without ground truth, but is expected to generalize well on many different target domains $X_{T}\subset {\mathbb{R}}^{H\times W\times 3}$. The convolutional autoencoder is composed of an encoder responsible for dimensionality reduction and a decoder responsible for dimensionality increase. The encoder function is represented by *Q*, and the decoder function is represented by *G*. Assuming that the encoder function gets the intermediate code *z*, the input image is represented by *x*_*s*_ ∈ *X*_*S*_, and the output image is represented by $x_{s^{+}}\in X_{S^{+}}$, then the entire encoder decoding process can be represented as:


(1) $$\begin{array}{l} \begin{array}{cc} \;z & =Q\left(x_{s}\right),\\ x_{s^{+}}=G\left(z\right) \end{array} \end{array}$$

Intuitively, we expect the augmented domain $X_{S^{+}}$ to be vastly different from the source domain *X*_*S*_ in vascular performance and brightness. In other words, we want to maximize the domain discrepancy between $X_{S^{+}}$ and *X*_*S*_. Therefore, the reconstruction error *L*_*CAE*_ for domain augmentation can be formulated as:


(2) $$L_{CAE}={\left\|x_{s}-x_{s^{+}}\right\|}^{2}$$

The pre-trained CAE can better capture the distribution of the source domain and maximize *L*_*CAE*_ creates large domain transportation.

The reason for choosing a convolutional autoencoder is that data slicing and data stacking can cause a large amount of information to be lost. The convolutional autoencoder abandons the stacked data, keeps its spatial information unchanged when the image data is input, and gently extracts the information in the convolutional layer. This process aims to preserve the spatial relationship in the data, but it does not generate the same data as GANs (Goodfellow et al., 2014).

### 2.2. Boundary and mask adversarial learning

The generalization of a model is directly related to the handling of boundaries. Therefore, to make the model more general, it needs to perform better in processing boundaries. We use adversarial learning on the boundaries and masks of the optic cup and optic disc, respectively. The core is to use the max-min game between the generator and the discriminator to obtain the optimal solution to determine whether it belongs to the enhanced source domain or the target domain. At the same time, boundary adversarial learning can be used to regress the boundary, so that the boundary prediction in the augmented domain is close to the source domain. Convolutional autoencoders then generate optic cups and optic discs that approximate the structured semantics of the source domain, but can represent different details (e.g., blurred vessel structures) in the augmentation domain. This method can further expand the source domain so that data from other domains can also perform well when training on the model, achieving the purpose of model generalization.

The source domain is $X_{S}\subset {\mathbb{R}}^{W\times H\times 3}$, which has segmentation labels made by professional ophthalmologists, denoted as $Y_{S}\subset {\mathbb{R}}^{W\times H}$ where *W* is the width of the picture and *H* is the height of the picture. And our enhanced domain generated by the convolutional autoencoder CAE is denoted as $X_{S^{+}}\subset {\mathbb{R}}^{W\times H\times 3}$. The source domain and the enhanced domain are mixed to become the enhanced source domain $X_{U}\subset {\mathbb{R}}^{W\times H\times 3}$. Since the source domain and the enhanced domain behave the same semantically, only the details are different, so the labels of the enhanced domain can use the labels corresponding to the source domain, and there is a one-to-one mapping relationship. For each image *x*_*s*_ ∈ *X*_*S*_ belonging to the source domain, an image $x_{s^{+}}\in X_{S^{+}}$ in the enhanced domain can be generated, and they share the label *y*_*s*_ ∈ *Y*_*S*_. When the source domain image is generated by CAE and becomes the enhanced domain, it will be put into our model together with the source domain image and the target domain image for training. The difference is, as shown in Figure 2, the images generated by the CAE module are processed with the first boundary discriminator to distinguish the source domain and the enhanced domain, the second mask discriminator to distinguish the new source domain (the union of the source domain and the enhanced domain) and the target domain, and the third boundary discriminator to distinguish the new source domain and the target domain. The three discriminators corresponding to the three branches will be described in detail below.

There are three discriminators with a different function for each. The first one is the discriminator $D_{b^{+}}$, which input is judged to belong to the source domain *X*_*S*_ (denoted as 1) or belongs to the enhancement domain $X_{S^{+}}$ (denoted as 0). This setting is to ensure that while the enhancement domain expands on the basis of the source domain, the boundary distribution of the enhancement domain approximates the boundary distribution of the source domain. Therefore, the training objective of the boundary discriminator between the enhancement domain and the source domain can be set as:


(3) $$L_{D_{b^{+}}}=\frac{1}{N}\left[\sum_{i\;=1}^{N}L_{D}\left(p_{x_{s}}^{b},1\right)+\sum_{i\;=1}^{N}L_{D}\left(p_{x_{s^{+}}}^{b},0\right)\right]$$

where N is the number of images in the source domain and the enhanced domain. Since the images in the enhanced domain and the source domain are generated with 1:1 (see Section 2.1), the number is N. And *L*_*D*_ is a common binary cross-entropy loss. At this time, in order to ensure that the boundary distribution of the enhancement domain will approximate the boundary distribution of the source domain, we need to further optimize the segmentation network:


(4) $$\begin{array}{l} L_{{adv}_{b^{+}}}=\frac{1}{N}\overset{N}{\underset{i\;=1}{\sum }}L_{D}\left(p_{x_{s^{+}}}^{b},1\right) \end{array}$$

The second one is the discriminator *D*_*b*_, input is judged to belong to the union *X*_*U*_ of the source domain *X*_*S*_ and the enhanced domain $X_{S^{+}}$ (marked as 1) or belong to the target domain *X*_*T*_ (marked as 0). Henceforth, this union *X*_*U*_ is called the new source domain. This setting is to ensure that the boundary distribution of the target domain is close to the boundary distribution of the new source domain, and to improve the prediction accuracy of the target domain in a boundary-driven manner. Therefore, the training objectives of the boundary discriminators of the new source and target domains can be set as:


(5) $$\begin{array}{l} L_{D_{b}}=\frac{1}{N_{U}}\overset{N_{U}}{\underset{i=1}{\sum }}L_{D}\left(p_{x_{u}}^{b},1\right)+\frac{1}{N_{T}}\overset{N_{T}}{\underset{i=1}{\sum }}L_{D}\left(p_{x_{t}}^{b},0\right) \end{array}$$

where *N*_*U*_ is the number of pictures in the new source domain, and *N*_*T*_ is the number of pictures in the target domain. At this time, in order to ensure that the boundary distribution of the target domain will approximate the boundary distribution of the new source domain, we still need to further optimize the segmentation network:


(6) $$\begin{array}{l} L_{{adv}_{b}}=\frac{1}{N_{T}}\overset{N_{T}}{\underset{i\;=1}{\sum }}L_{D}\left(p_{x_{t}}^{b},1\right) \end{array}$$

Finally, there is a discriminator *D*_*m*_. If only boundary-driven confrontation is used, it is still easy to generate uncertain predictions inside the optic cup and optic disc, resulting in an only good performance at the boundary. However, the goal is to segment the entire optic cup and the optic disc. Therefore, we also need to perform adversarial learning on masks to narrow the distribution difference between the new source and target domains. Specifically, the objectives of our mask discriminator for new source and target domains can be set as:


(7) $$\begin{array}{l} L_{D_{m}}=\frac{1}{N_{U}}\overset{N_{U}}{\underset{i\;=1}{\sum }}L_{D}\left(p_{x_{u}}^{m},1\right)+\frac{1}{N_{T}}\overset{N_{T}}{\underset{i\;=1}{\sum }}L_{D}\left(p_{x_{t}}^{m},0\right) \end{array}$$

At the same time, we still need to optimize the segmentation network and perform adversarial learning to fool the discriminator and make the target domain images generate prediction masks close to the new source domain. The detailed settings are as follows:


(8) $$\begin{array}{l} L_{{adv}_{m}}\;=\frac{1}{N_{T}}\overset{N_{T}}{\underset{i\;=1}{\sum }}L_{D}\left(p_{x_{t}}^{m},1\right) \end{array}$$

Combine *L*_*adv*_*m*__, *L*_*adv*_*b*__, and $L_{{adv}_{b^{+}}}$ as:


(9) $$\begin{array}{l} L_{adv}={L_{{adv}_{m}}+L}_{{adv}_{b}}+L_{{adv}_{b^{+}}} \end{array}$$

### 2.3. Network architecture and loss function

As it is shown in Figure 2, the backbone is based on the DeepLabv3+ (Chen et al., 2018) architecture and adds the Atrous Spatial Pyramid Pooling (ASPP) component to capture contextual information at multiple scales. The high-level and low-level features are then concatenated, and the boundary and mask prediction branches are added after this. Both the boundary prediction branches are composed of the same structure, which is composed of three convolutional layers, and the output channels are 256, 256, and 1, respectively. The input of the mask branch is the concatenation of the shared features and the boundary prediction. The advantage of this design lies in the ability to bound the fine-grained segmentation masks with the help of boundary supervision.

For the specific structure of the convolutional autoencoder part, see Section 2.1. The last part is the discriminator. The three discriminators $D_{b^{+}}$, *D*_*b*_ and *D*_*m*_ have a similar structure, which contains five convolutional layers, and the number of channels is 64, 128, 256, 512, 1 and in that order. And the kernel size of these five layers is 4 × 4, and the stride size is 2 × 2. After each convolutional layer, there is a LeakyReLU activation function instead of the ReLU (α = 2). But the last layer is special, using the Sigmoid activation function. Through recursive iteration, the receptive field size of each discriminator is 94 × 94. Subsequently, each patch output size is 16 × 16 and is distinguished as true (1) or false (0) by $D_{b^{+}}$, *D*_*b*_ and *D*_*m*_.

In the loss function, *L*_*m*_ is the mask prediction loss, which takes the form of cross-entropy and performs multi-label classification processing (Wang et al., 2019b) simultaneously, so that the optic cup and optic disc can be segmented at the same time. *L*_*b*_ is the boundary regression loss. The loss functions *L*_*m*_ and *L*_*b*_ are:


(10) $$\begin{array}{l} L_{m}=-\frac{1}{N}\sum_{i=1}^{N}[y_{x_{u}}^{m}\cdot log(p_{x_{u}}^{m})+(1-y_{x_{u}}^{m})\cdot log(1-p_{x_{u}}^{m})],\\ L_{b}=\frac{1}{N}\sum_{i=1}^{N}(y_{x_{u}}^{b}-p_{x_{u}}^{b}{)}^{2} \end{array}$$

where *p* ∈ [0, 1] represents the predicted probability and *y* ∈ {0, 1} represents the ground truth of the segmentation.

Finally, the overall segmentation network loss is:


(11) $$\begin{array}{l} L=L_{m}+L_{b}+\lambda_{adv}L_{adv} \end{array}$$

where λ_*adv*_ is the parameter used to balance the loss. The discriminator is then trained according to Equations (3), (5), and (7).

## 3. Experiments and results

### 3.1. Experimental setup

We evaluate the proposed method with three public available datasets REFUGE challenge dataset (Orlando et al., 2020), Drishti-GS (Sivaswamy et al., 2015) and RIM-ONE-r3 (Fumero et al., 2011). Since only the optic cup and optic disc are segmented, we processed the original dataset and re-cropped it into ROIs (Wang et al., 2019b) of size 512 × 512, centered on the OD. Following existing experience (Wang et al., 2019b), we apply standard data augmentation strategies to the images, including Gaussian noise, random erasure, rotation, contrast adjustment, elastic transformation, etc. The detailed statistics of the dataset are shown in Table 2.

**Table 2:** Public fundus datasets used for training and testing in experiments.

**Domain**	**Dataset**	**Number of samples (train+test)**	**Scanners**
Source	REFUGE(train)	320 + 80	Zeiss Visucam 500
Target	Drishti-GS	50 + 51	(Aravind eye hospital)
Target	RIM-ONE-r3	99 + 60	Nidek AFC-210
Target	REFUGE(val)	320 + 80	Canon CR-2

We implement CAE-BMAL in Pytorch^1^. The source and enhanced domain boundary discriminator $D_{b^{+}}$, the source and target domain boundary discriminator *D*_*b*_ and the mask discriminator *D*_*m*_ are all optimized by the stochastic gradient descent SGD algorithm, and the initial learning rate is 1e-3 according to experience. The segmentation network is optimized by the Adam algorithm, and the learning rate is set to 2.5e-5 at this time. We train on a GPU server with a single NVIDIA TESLA V100 32G.

### 3.2. Evaluation indicators

We adopt the Dice Similarity Coefficient (DSC) and the Vertical Cup-to-Disk Ratio (CDR) to evaluate the segmentation performance. Among them, DSC and CDR are defined as:


(12) $$\begin{array}{l} \;DSC=\frac{2\times TP}{2\times TP+FP+FN},\\ \;\delta =\left|CDR_{p}-CDR_{g}\right|,\\ CDR=\frac{d(OC)}{d(OD)} \end{array}$$

where *TP*, *TN*, *FP*, and *FN* represent the number of true positives, true negatives, false positives, and false negatives at the pixel level, respectively. *d*(*OC*) and *d*(*OD*) represent the vertical diameters of OC and OD and are used to represent the error between the predicted cup-to-disk ratio and the cup-to-disk ratio given by the actual clinician. We want the bigger the *DSC* the better and the smaller the better. The reason why we choose these two evaluation indicators is that the dice coefficient is usually a commonly used evaluation indicator for segmentation tasks, especially for optic cup and optic disc segmentation tasks, and the *CDR* value is one of the important indicators used in glaucoma screening in clinical medicine.

### 3.3. Performance on Drishti-GS and RIM-ONE-r3 datasets

**Figure 3:**
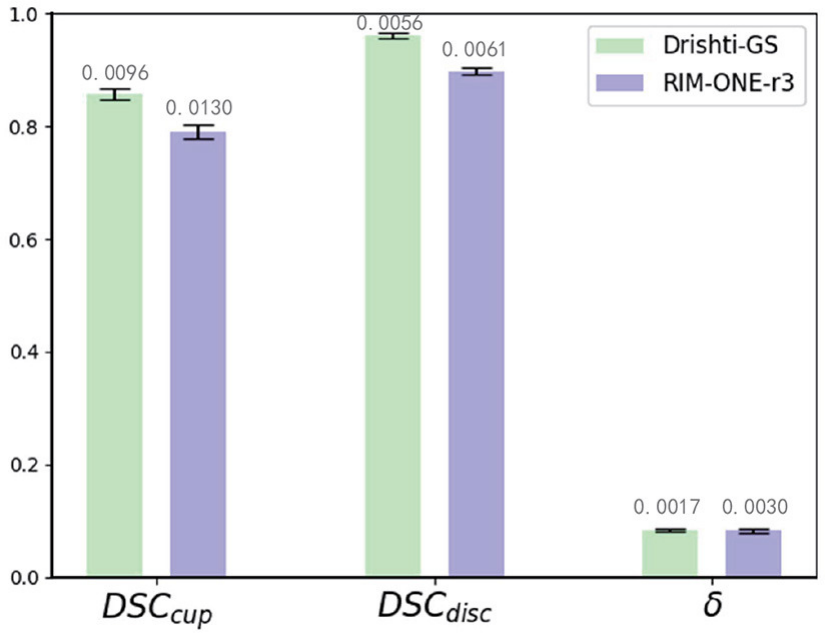
Mean performance and standard deviation on the Drishti-GS dataset and the RIM-ONE-r3 dataset.

We compare our method with state-of-the-art domain adaptation or domain generalization segmentation methods: BEAL (Wang et al., 2019a), DoFE (Wang S. et al., 2020), CADA (Liu et al., 2022), CFEA (Liu et al., 2019), AdaptSegNet (Tsai et al., 2018), DAE (Karani et al., 2021), and SRDA (Bateson et al., 2020) for comparison. We use REFUGE (train) as the source domain and Drishti-GS (train), and RIM-ONE-r3 (train) as the target domain for training, and tested with Drishti-GS (test) and RIM-ONE-r3 (test), respectively. For BEAL, DoFE, CADA and CFEA, we follow the parameters mentioned in these paper and evaluate the performance. For AdaptSegNet, DAE and SRDA, we follow the parameters mentioned in these paper but replace the evaluation metrics to implement the experiments on REFUGE, Drishti-GS and RIM-ONE-r3 datasets. Then all these 7 state-of-the-art are evaluated with the proposed MAE-BMAL method under same metric. The quantitative results are shown in Table 3. It can be seen that the proposed CAE-BMAL method outperforms other domain-adaptive comparison methods on the OD segmentation metrics, which can indicate that our framework has a stronger generalization ability. Although the BEAL method performs better than our method on OC segmentation, it does not evaluate the CDR and therefore cannot screen for glaucoma. And more importantly, due to the influence of light, the OC boundary itself is more difficult to discriminate than the OD boundary of most pictures, and our method partly relies on boundary discrimination. Compared with other methods, our method is at least 2.8% lower in Drishti-GS, and the performance is significantly improved; it is also reduced by at least 0.5% in RIM-ONE-r3, so it can play a better role in the practical application of glaucoma discrimination. Meanwhile, we run the experiment 5 times and report the average performance and standard deviation, as shown in Figure 3. The number on each bar is the standard deviation, the three green bars are the results on the Drishti-GS dataset, and the three blue bars are the results on the RIM-ONE-r3 dataset. The unit of the y-axis is the same as that in Table 3, and the height of each column corresponds to the result of the Ours row in Table 3, that is, the average value after 5 experiments.

**Table 3:** Comparison of segmentation methods using domain adaptation.

**Methods**	**Drishti-GS dataset**			**RIM-ONE-r3 dataset**		
	***DSC*_*cup*_**	***DSC*_*disc*_**	**δ**	***DSC*_*cup*_**	***DSC*_*disc*_**	**δ**
BEAL (Wang et al., 2019a)	**0.862**	0.961	-	**0.810**	0.898	-
DoFE (Wang S. et al., 2020)	0.835	0.955	-	0.800	0.893	-
CADA (Liu et al., 2022)	0.840	0.890	0.111	0.640	0.766	0.087
CFEA (Liu et al., 2019)	0.827	0.887	0.113	0.635	0.751	0.095
AdaptSegNet (Tsai et al., 2018)	0.826	0.881	0.118	0.627	0.737	0.102
DAE (Karani et al., 2021)	0.831	0.940	-	0.790	0.891	-
SRDA (Bateson et al., 2020)	0.807	0.962	-	0.776	0.894	-
Ours	0.857	**0.962**	**0.083**	0.791	**0.898**	**0.082**

The best result of the column is shown in bold.

**Figure 4:**
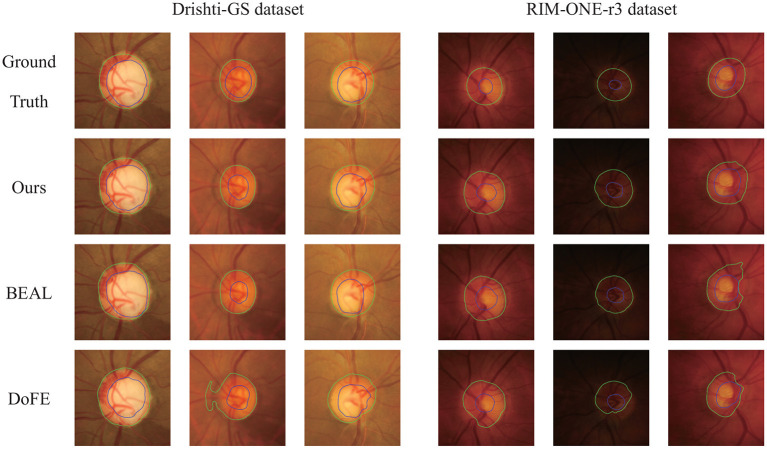
Qualitative segmentation results were trained on REFUGE and tested on Drishti-GS and RIM-ONE-r3 datasets. The samples were randomly selected from their respective datasets. Green and blue outlines represent the boundaries of OD and OC, respectively.

Figure 4 shows several qualitative segmentation results of the proposed method compared to BEAL and DoFE. To ensure the fairness of the experiment, we use the same scale to crop the ROI region. As can be seen from the visual contours, the segmentation results of our scheme under various lighting conditions (especially low-light conditions) are better and closer to the ground truth than the other two.

**Figure 5:**
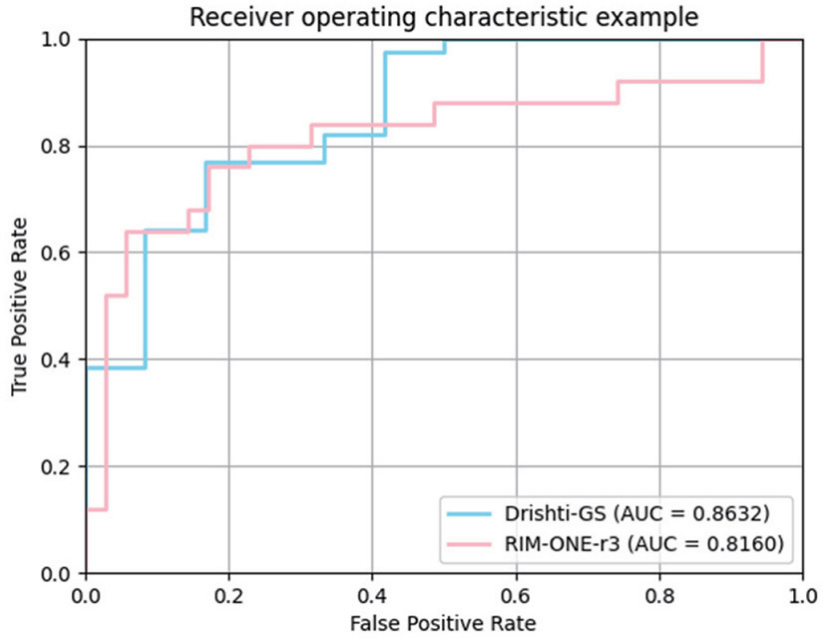
ROC curves of our method for glaucoma screening on Drishti-GS and RIM-ONE-r3 datasets.

In the medical field, the vertical CDR value is one of the important indicators for glaucoma screening. To verify the correlation between the CDR estimates derived from our model and glaucoma, we further plotted ROC curves on the Drishti-GS and RIM-ONE-r3 datasets. The curve is obtained by calculating the true positive rate and false positive rate by setting different thresholds. The closer the ROC curve is to the upper left corner, the better the performance. AUC (Area Under Curve) is defined as the area under the ROC curve. The larger the area, the better the effect. We plotted the ROC curve in Figure 5 and calculated the AUC.

### 3.4. Performance on REFUGE dataset

We combine the optic cup and optic disc segmentation results of the MICCAI 2018 report REFUGE challenge and use the same dataset. The challenge is weighted according to the following formula, and the individual and total scores are ranked:


(13) $$\begin{array}{l} R=0.35\times R_{cup}+0.25\times R_{disc}+0.4\times R_{\delta } \end{array}$$

A total of 12 teams were selected for this challenge^2^, to which we compared our results and redrawn Table 4. To further improve the segmentation performance, the participating teams ensemble several models to obtain predictions on the images of the test set. For example, the CUHKMED team integrated five models. In contrast, we only took the form of a single model and ranked fourth overall in the absence of third-party data to support it. At the same time, our model achieves the best results on optic disc segmentation, ranking first, and improves the corresponding term by 0.26%; it also achieves good results on optic disc segmentation, ranking third. However, due to the unclear boundary between the optic cup and the optic disc caused by the light, the boundary of the optic cup segmentation is not smooth enough, which further leads to poor performance compared with other teams. Later, we consider adding other attention modules to specifically deal with the cup. But overall, our model achieves good results in dealing with segmentation under the influence of complex blood vessels.

**Table 4:** Cup/disc segmentation results on the REFUGE test set.

**Overall rank**	**Team**	**Score**	***DSC*_*cup*_**	***R*_*DS*_*C*__*cup*__**	***DSC*_*disc*_**	***R*_*DS*_*C*__*disc*__**	**δ**	***R*_δ_**
**1**	**CUHKMED**	**1.75**	0.8826	2	0.9602	2	0.0450	2
2	Masker	2.50	**0.8837**	**1**	0.9464	8	**0.0414**	**1**
3	BUCT	3.00	0.8728	4	0.9525	4	0.0456	3
4	Ours	4.10	0.8786	3	**0.9628**	**1**	0.0520	7
5	NKSG	4.60	0.8643	6	0.9488	6	0.0465	4
6	VRT	5.40	0.8600	7	0.9532	3	0.0525	8
7	AIML	5.45	0.8519	8	0.9505	5	0.0469	5
8	Mammoth	7.10	0.8667	5	0.9361	11	0.0526	9
9	SMILEDeepDR	7.45	0.8367	9	0.9386	10	0.0488	6
10	NIGHTOwl	8.60	0.8257	11	0.9487	7	0.0563	10
11	SDSAIRC	9.15	0.8315	10	0.9436	9	0.0674	11
12	Cvblab	11.00	0.7728	12	0.9077	12	0.0798	12
13	Winter_ Fell	12.00	0.6861	13	0.8772	13	0.1536	13

The best result of the column is shown in bold.

### 3.5. Ablation studies

In this section, we discuss the performance of each part on the REFUGE dataset and its impact. We use the backbone based on DeepLabv3+ and the architecture with Atrous Spatial Pyramid Pooling (ASPP) components as the baseline. We added CAE, BMAL, and $D_{b^{+}}$ to the baseline to verify the necessity of these modules. Among them, CAE and $D_{b^{+}}$ are in order, because only by adding the CAE module can the discriminator $D_{b^{+}}$ of the source domain and the enhanced domain be further added. Therefore, it is not possible to add $D_{b^{+}}$ after the baseline to verify the necessity alone. It is also worth noting that BMAL here refers to two branches that do not include $D_{b^{+}}$: the mask and boundary discriminant branches, which are a combination. Table 5 presents the quantitative results for various combinations. It can be seen that each additional module in our model is improved on this basis, that is, each module is essential. We also found the following: (1) For the two indicators *DSC*_*cup*_ and *DSC*_*disc*_, the BMAL has a higher improvement than the CAE module. However, for the δ, CAE improves more than the BMAL module; (2) After combining the two modules, there is a common improvement in the three indicators, especially δ is reduced by 1.59%; (3) At the same time, adding $D_{b^{+}}$ on this basis will further improve the performance. The main reason is that CAE is to increase the distribution between the source domain and the enhancement domain, and the discriminator is to narrow the distribution of each other. Such a setting can make the model improve the generalization ability as much as possible without losing the segmentation performance; (4) Although δ is not only determined by *DSC*_*cup*_ and *DSC*_*disc*_, the accurate segmentation of OC and OD does help to estimate the value of δ.

**Table 5:** Ablation experiments on the REFUGE test set.

**Methods**				**REGUSE dataset**		
**Baseline**	**CAE**	**BMAL w/o $D_{b^{+}}$**	** $D_{b^{+}}$ **	***DSC*_*cup*_**	***DSC*_*disc*_**	**δ**
✓				0.8427	0.9387	0.0785
✓	✓			0.8526	0.9425	0.0702
✓		✓		0.8652	0.9514	0.0721
✓	✓	✓		0.8725	0.9610	0.0562
✓	✓	✓	✓	**0.8786**	**0.9628**	**0.0520**

The best result of the column is shown in bold.

**Figure 6:**
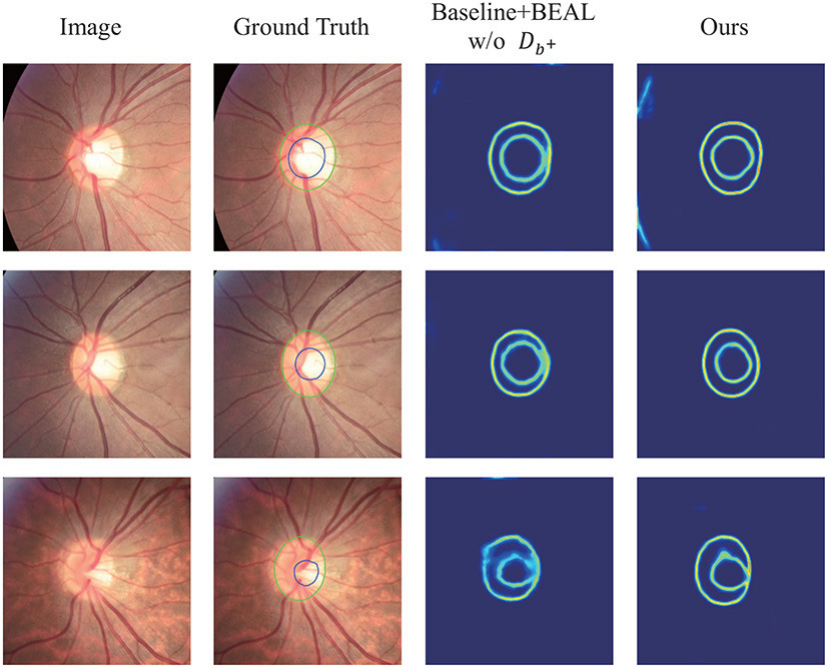
Testing the impact of the overall CAE module on the boundary prediction map on REFUGE.

By observing Figure 6, it can be concluded that after adding CAE and $D_{b^{+}}$ to our network, the boundary discrimination is clearer, which further improves the accuracy of segmentation and reduces the error of δ.

### 3.6. Comparison of other backbone networks

This paper compares the three backbone networks of ResNet-34 (He et al., 2016), DenseNet (Huang et al., 2017), and MobileNetV2 (Sandler et al., 2018). We fully consider the advantages and disadvantages of the three models and conduct comparative experiments.

Four different backbone networks are used to evaluate, including ResNet-34, DenseNet, MobileNetV2 and Deeplabv3+. We fully consider the advantages and disadvantages of the four models and conduct comparative experiments.

The experimental results are shown in Table 6. It exhibits that when other modules remain unchanged, the DSC of the original backbone MobileNetV2 on Drishti-GS is 0.834 and 0.948, respectively, and Deeplabv3+ is increased by 2.3% and 1.4%, and the δ also decreased by 1.6%. The DSC on the RIM-ONE-r3 increased by 1.9% and 1.8%, respectively, while the δ decreasing by 2.0%. For DenseNet, it is obvious that the significant performance decline is δ, due to a reduce in the number of network layers, which result in a lose of long-term feature dependencies. Although the performance of ResNet is similar to that of Deeplabv3+, due to the large network model, it is very time-consuming to process medical images.

**Table 6:** Comparison of ResNet, DenseNet, MobileNetV2, and DeepLabv3+ as backbone.

**Methods**	**Drishti-GS dataset**			**RIM-ONE-r3 dataset**		
	***DSC*_*cup*_**	***DSC*_*disc*_**	**δ**	***DSC*_*cup*_**	***DSC*_*disc*_**	**δ**
ResNet (He et al., 2016)	0.849	**0.962**	0.089	0.875	0.895	0.083
DenseNet (Huang et al., 2017)	0.831	0.954	0.122	0.782	0.892	0.126
MobileNetV2 (Sandler et al., 2018)	0.834	0.948	0.099	0.772	0.880	0.102
DeepLabv3+ (Chen et al., 2018)	**0.857**	**0.962**	**0.083**	**0.791**	**0.898**	**0.082**

The best result of the column is shown in bold.

## 4. Discussions and conclusion

The Cup-to-disc ratio has been recognized as an essential attribute of glaucoma screening, thus the automated and accurate segmentation of optic cup and the optic disc has become the subject of widespread research. Although there has been a lot of previous work on this problem, problems such as sparse labels or different clinical image distributions lead to domain shifts in the training and test sets. In other words, it may lead to a significant gap between the researched model and the actual clinical application. To this end, we propose a fundus image segmentation method based on unsupervised domain adaptation, which makes the target domain prediction approach the source domain prediction, so that the optic cup and optic disc segmentation can be more widely used in clinical glaucoma screening and diagnosis. We conduct cross-domain studies and extensive experiments on three datasets, Drishti-GS, RIM-ONE-r3, and REFUGE, and the results show that our method significantly outperforms other domain transfer methods.

In our segmentation method, we employ the same preprocessing as in previous work, i.e., use an extraction network to crop the ROI image before performing segmentation (Wang et al., 2019b). Our framework augments the source domain with a convolutional autoencoder to generate an augmented domain with the same semantics as the source domain, but with different vessel structures and degrees of lightness and darkness. At the same time, we combine multiple adversarial learning discriminators to learn the boundaries and masks of the source domain, the enhanced domain, and the target domain respectively, especially to solve the problem of inaccurate segmentation due to blurred boundaries caused by the influence of blood vessels and light.

Although our method has obvious improvements in fundus image segmentation, it still suffers from some limitations. First, our segmentation method performs significantly better on optic disc segmentation than optic cup segmentation. The reason is that affected by the light and the color of the fundus itself, the boundary of the optic cup is more blurred, resulting in ambiguity in pixel-level discrimination. A potential solution is to use a mask to selectively perform further fine-grained learning on the pixels around the predicted boundary of the optic cup. Second, although the distribution of images in the same domain is roughly the same, there are also cases where different types of subdomains exist. Domain augmentation at the subdomain level may further improve the accuracy of segmentation results. However, a good solution has not yet been found to divide the subdomain. Unsupervised clustering (Caron et al., 2018) may be one of the solutions to the subdomain problem. Future work will explore its possibility and integrate it into our model. Besides, we only conduct experiments on fundus segmentation tasks, and we will generalize our framework to other medical image segmentation tasks in the future to further demonstrate the effectiveness and generality of our framework.

## Data availability statement

Publicly available datasets were analyzed in this study. This data can be found at: (Drishti-GS) http://cvit.iiit.ac.in/projects/mip/drishti-gs/mip-dataset2/enter.php; (RIM-ONE-r3) http://medimrg.webs.ull.es/research/downloads/; (REFUGE) (Grand Challenge) https://refuge.grand-challenge.org/.

## Author contributions

XZ: conceptualization, supervision, writing, and review and editing. JS: conceptualization, methodology, writing, data acquisition, analysis, and interpretation. CW: validation and visualization. ZZ: data acquisition, data curation, validation, and visualization. All authors contributed to the article and approved the submitted version.

## Funding

This research was supported in part by the Natural Science Foundation of Chongqing, China (No. cstc2019jscx-mbdxX0021), in part by the Project of Key Laboratory of Tourism Multisource Data Perception and Decision (Ministry of Culture and Tourism) and in part by the Key Cooperation Project of Chongqing Municipal Education Commission (No. HZ2021008).

## Conflict of interest

Author CW was employed by Chongqing Telecom System Integration Co., Ltd., Chongqing, China. The remaining authors declare that the research was conducted in the absence of any commercial or financial relationships that could be construed as a potential conflict of interest.

## Publisher's note

All claims expressed in this article are solely those of the authors and do not necessarily represent those of their affiliated organizations, or those of the publisher, the editors and the reviewers. Any product that may be evaluated in this article, or claim that may be made by its manufacturer, is not guaranteed or endorsed by the publisher.

## Footnotes

^1^ https://github.com/CQRhinoZ/CAE-BMAL

^2^ https://refuge.grand-challenge.org/Results-Onsite_TestSet/

## References

[B1] Bateson M. Kervadec H. Dolz J. Lombaert H. Ayed I. B. (2020). Source-relaxed domain adaptation for image segmentation, in Medical Image Computing and Computer Assisted Intervention–MICCAI 2020–23rd International Conference, Lima, Peru, October 4-8, 2020, Proceedings, Part I, volume 12261 of Lecture Notes in Computer Science, eds Martel A. L. Abolmaesumi P. Stoyanov D. Mateus D. Zuluaga M. A. Zhou S. K. Racoceanu D. Joskowicz L. (Lima: Springer), 490–499.

[B2] Caron M. Bojanowski P. Joulin A. Douze M. (2018). Deep clustering for unsupervised learning of visual features, in Computer Vision–ECCV 2018–15th European Conference, Munich, Germany, September 8-14, 2018, Proceedings, Part XIV, volume 11218 of Lecture Notes in Computer Science, eds Ferrari V. Hebert M. Sminchisescu C. Weiss Y. (Munich: Springer), 139–156.

[B3] Chen C. Bai W. Davies R. H. Bhuva A. N. Manisty C. H. Augusto J. B.. (2020). Improving the generalizability of convolutional neural network-based segmentation on cmr images. Front. Cardiovasc. Med. 7, 105. 10.3389/fcvm.2020.00105 32714943 PMC7344224

[B4] Chen L. Zhu Y. Papandreou G. Schroff F. Adam H. (2018). Encoder-decoder with atrous separable convolution for semantic image segmentation, in Computer Vision–ECCV 2018–15th European Conference, Munich, Germany, September 8-14, 2018, Proceedings, Part VII, volume 11211 of Lecture Notes in Computer Science, eds Ferrari V. Hebert M. Sminchisescu C. Weiss Y. (Munich: Springer), 833–851.

[B5] Dou Q. Ouyang C. Chen C. Chen H. Heng P. A. (2018). Unsupervised cross-modality domain adaptation of convnets for biomedical image segmentations with adversarial loss, in International Joint Conference on Artificial Intelligence (Stockholm), 691–697.

[B6] Fu H. Cheng J. Xu Y. Wong D. W. K. Liu J. Cao X. (2018). Joint optic disc and cup segmentation based on multi-label deep network and polar transformation. IEEE Trans. Med. Imaging 37, 1597–1605. 10.1109/TMI.2018.2791488 29969410

[B7] Fumero F. Alayón S. Sánchez J. L. Sigut J. F. González-Hernández M. (2011). RIM-ONE: an open retinal image database for optic nerve evaluation, in 2011 24th International Symposium on Computer-Based Medical Systems (CBMS) (Bristol: IEEE).

[B8] Goodfellow I. J. Pouget-Abadie J. Mirza M. Xu B. Warde-Farley D. Ozair S.. (2014). Generative adversarial nets, in Advances in Neural Information Processing Systems 27: Annual Conference on Neural Information Processing Systems 2014, December 8-13 2014, Montreal, Quebec, Canada, eds Ghahramani Z. Welling M. Cortes C. Lawrence N. D. Weinberger K. Q. (Montreal, QC), 2672–2680.

[B9] He K. Zhang X. Ren S. Sun J. (2016). Deep residual learning for image recognition, in 2016 IEEE Conference on Computer Vision and Pattern Recognition, CVPR 2016 (Las Vegas, NV: IEEE Computer Society), 770–778.

[B10] Huang G. Liu Z. van der Maaten L. Weinberger K. Q. (2017). Densely connected convolutional networks, in 2017 IEEE Conference on Computer Vision and Pattern Recognition, CVPR 2017 (Honolulu, HI: IEEE Computer Society), 2261–2269.

[B11] Karani N. Erdil E. Chaitanya K. Konukoglu E. (2021). Test-time adaptable neural networks for robust medical image segmentation. Med. Image Anal. 68, 101907. 10.1016/j.media.2020.101907 33341496

[B12] Liu P. Kong B. Li Z. Zhang S. Fang R. (2019). CFEA: collaborative feature ensembling adaptation for domain adaptation in unsupervised optic disc and cup segmentation, in Medical Image Computing and Computer Assisted Intervention–MICCAI 2019–22nd International Conference, Shenzhen, China, October 13-17, 2019, Proceedings, Part V, volume 11768 of Lecture Notes in Computer Science, eds Shen D. Liu T. Peters T. M. Staib L. H. Essert C. Zhou S. Yap P. Khan A. R. (Shenzhen: Springer), 521–529.

[B13] Liu P. Tran C. T. Kong B. Fang R. (2022). CADA: multi-scale collaborative adversarial domain adaptation for unsupervised optic disc and cup segmentation. Neurocomputing 469, 209–220. 10.1016/j.neucom.2021.10.076

[B14] Orlando J. I. Fu H. Breda J. B. van Keer K. Bathula D. R. Diaz-Pinto A.. (2020). REFUGE challenge: A unified framework for evaluating automated methods for glaucoma assessment from fundus photographs. Med. Image Anal. 59, 101570. 10.1016/j.media.2019.101570 31630011

[B15] Prakash A. Boochoon S. Brophy M. Acuna D. Cameracci E. State G.. (2019). Structured domain randomization: bridging the reality gap by context-aware synthetic data, in International Conference on Robotics and Automation, ICRA 2019 (Montreal, QC: IEEE), 7249–7255.

[B16] Sandler M. Howard A. G. Zhu M. Zhmoginov A. Chen L. (2018). Mobilenetv2: inverted residuals and linear bottlenecks, in 2018 IEEE Conference on Computer Vision and Pattern Recognition, CVPR 2018 (Salt Lake City, UT: Computer Vision Foundation; IEEE Computer Society), 4510–4520.

[B17] Sivaswamy J. Krishnadas S. Chakravarty A. Joshi G. D. Ujjwal Syed, T. A. (2015). A comprehensive retinal image dataset for the assessment of glaucoma from the optic nerve head analysis. JSM Biomed. Imag. Data Papers. 2, 1004. Available online at: http://cdn.iiit.ac.in/cdn/cvit.iiit.ac.in/images/ConferencePapers/2015/Arunava2015AComprehensive.pdf

[B18] Tsai Y. Hung W. Schulter S. Sohn K. Yang M. Chandraker M. (2018). Learning to adapt structured output space for semantic segmentation, in 2018 IEEE Conference on Computer Vision and Pattern Recognition, CVPR 2018 (Salt Lake City, UT: Computer Vision Foundation; IEEE Computer Society), 7472–7481.

[B19] Wang S. Yu L. Li K. Yang X. Fu C. Heng P. (2020). Dofe: Domain-oriented feature embedding for generalizable fundus image segmentation on unseen datasets. *IEEE Trans*. Med. Imaging 39, 4237–4248. 10.1109/TMI.2020.3015224 32776876

[B20] Wang S. Yu L. Li K. Yang X. Fu C.-W. Heng P.-A. (2019a). Boundary and entropy-driven adversarial learning for fundus image segmentation, in International Conference on Medical Image Computing and Computer-Assisted Intervention (Shenzhen: Springer), 102–110.

[B21] Wang S. Yu L. Yang X. Fu C. Heng P. (2019b). Patch-based output space adversarial learning for joint optic disc and cup segmentation. IEEE Trans. Med. Imaging 38, 2485–2495. 10.1109/TMI.2019.2899910 30794170

[B22] Wang Z. Keane P. A. Chiang M. Cheung C. Y. Wong T. Y. Ting D. S. W. (2020). Artificial intelligence and deep learning in ophthalmology, in Artificial Intelligence in Medicine, eds Lidströmer N. Ashrafian H. (Cham: Springer).

[B23] Yue X. Zhang Y. Zhao S. Sangiovanni-Vincentelli A. L. Keutzer K. Gong B. (2019). Domain randomization and pyramid consistency: Simulation-to-real generalization without accessing target domain data, in 2019 IEEE/CVF International Conference on Computer Vision, ICCV 2019 (Seoul: IEEE), 2100–2110.

[B24] Zaaboub N. Sandid F. Douik A. Solaiman B. (2022). Optic disc detection and segmentation using saliency mask in retinal fundus images. Comput. Biol. Med. 150, 106067. 10.1016/j.compbiomed.2022.106067 36150251

[B25] Zhang Y. Miao S. Mansi T. Liao R. (2018). Task driven generative modeling for unsupervised domain adaptation: application to x-ray image segmentation, in Medical Image Computing and Computer Assisted Intervention–MICCAI 2018–21st International Conference, Granada, Spain, September 16-20, 2018, Proceedings, Part II, volume 11071, eds Frangi A. F. Schnabel J. A. Davatzikos C. Alberola-López C. Fichtinger G. (Granada: Springer), 599–607.

[B26] Zhang Z. Li Y. Shin B.-S. (2022). C2-gan: content-consistent generative adversarial networks for unsupervised domain adaptation in medical image segmentation. Med. Phys. 49, 6491–6504. 10.1002/mp.15944 35981348

[B27] Zhou K. Yang Y. Hospedales T. M. Xiang T. (2020). Deep domain-adversarial image generation for domain generalisation, in The Thirty-Fourth AAAI Conference on Artificial Intelligence, AAAI 2020, The Thirty-Second Innovative Applications of Artificial Intelligence Conference, IAAI 2020, The Tenth AAAI Symposium on Educational Advances in Artificial Intelligence, EAAI 2020 (New York, NY: AAAI Press), 13025–13032.

